# The complete mitochondrial genomes of the higher termites *Labiotermes labralis* and *Embiratermes neotenicus* (Termitidae: Syntermitinae)

**DOI:** 10.1080/23802359.2017.1289349

**Published:** 2017-02-21

**Authors:** Vincent Hervé, Andreas Brune

**Affiliations:** Department of Biogeochemistry, Max Planck Institute for Terrestrial Microbiology, Marburg, Germany

**Keywords:** Termite, mitogenome, Isoptera, Termitidae, Syntermitinae

## Abstract

The complete mitochondrial genomes (mitogenomes) of two higher termites were reconstructed from the metagenomes of individual gut compartments with more than 2000-fold coverage. The circular mitogenomes of *Labiotermes labralis* (accession number KY436201) and *Embiratermes neotenicus* (accession number KY436202) have a length of 15,935 and 15,868 bp and an overall G + C content of 30.7 mol% and 33.7 mol%, respectively, and both have an asymmetric nucleotide composition. Genome structure and orientation are the same as in other termites and in the ancestral insect mitogenome. These data further expand arthropod mitogenome databases, which have become an important resource in ecological, phylogenetic and evolutionary studies.

Over the last few years, mitochondrial genomes (mitogenomes) have received strong interest by both evolutionary biologists and ecologists (Crampton-Platt et al. 2016). The number of available arthropod mitogenomes, especially from insects, has greatly increased during the last decade (Cameron 2014). The genomic information contained in these mitogenomes not only allows inference of phylogeny and evolutionary history of insects, such as termites, with a unprecedented resolution (Bourguignon et al. 2015), but also provides a phylogenetic framework for monitoring arthropod biodiversity in soils with high-throughput DNA sequencing approaches (Andújar et al. 2015; Crampton-Platt et al. 2015).

Termites are key organisms in tropical ecosystems because they are important soil engineers (Jouquet et al. 2016) and play a significant role in lignocellulose degradation (Brune 2014). Here we report the complete mitochondrial genome sequences of two species of higher termites (Termitidae: Syntermitinae), *Labiotermes labralis* and *Embiratermes neotenicus*, which were reconstructed from the metagenomic reads of the insect guts. Termites were collected in Petit Saut, French Guiana. The DNA of both samples is stored at the Max Planck Institute of Terrestrial Microbiology, Marburg, Germany.

The mitogenomes were reconstructed using a strategy that has proven to be efficient for other termite species (Dietrich & Brune 2016; Qian 2016). Metagenomes of individual gut compartments of *Labiotermes labralis* (segment P1; IMG taxon object ID 3300010369) and *Embiratermes neotenicus* (segment P4; IMG taxon object ID 3300009784) were obtained as previously described (Rossmassler et al. 2015). The quality-trimmed sequencing reads from the gut metagenomes were collected, and mitochondrial reads were extracted and assembled using a baiting and iterative mapping approach (MITObim version 1.8; Hahn et al. 2013) with the complete mitogenome of *Nasutitermes triodiae* (accession number JX144940) as reference genome. The resulting mitogenomes were circularized and then annotated with the Improved *de novo* Metazoan Mitochondrial Genome Annotation (MITOS) webserver (Bernt et al. 2013; http://mitos.bioinf.uni-leipzig.de/).

The original metagenomic dataset of *Labiotermes labralis* consisted of 191,583,421 raw reads, from which 244,794 mitochondrial reads were recruited and assembled into one circular contig of 15,935 bp. The mitogenome sequence had an average coverage of 2023-fold, an asymmetric nucleotide composition (42.9% A, 19.2% C, 11.4% G, 26.5% T) and an overall G + C content of 30.7 mol% (accession number KY436201). The original metagenomic dataset of *Embiratermes neotenicus* consisted of 209,047,724 raw reads, from which 269,723 mitochondrial reads were recruited and assembled into one circular contig of 15,868 bp, with an average coverage of 2418-fold, an asymmetric nucleotide composition (42.5% A, 21.6% C, 12.1% G, 23.8% T) and an overall G + C content of 33.7 mol% (accession number KY436202). These values are consistent with the length and nucleotide composition of other higher termite mitogenomes (Bourguignon et al. 2015; Dietrich & Brune 2016).

Both mitogenomes encode 37 genes including 2 ribosomal RNA (rRNA) genes (12S and 16S rRNA), 22 transfer RNA (tRNA) genes, and 13 protein-coding genes, and also contain a non-coding control region. The protein-coding genes encode seven NADH dehydrogenase subunits (*nad1-nad6, nad4l*), the cytochrome *b* subunit (*cob*), three cytochrome *c* oxidase subunits (*cox1–cox3*), and two ATP synthase subunits (*atp6, atp8*). In both cases, the gene order and orientation were identical to those of other termites, which have retained the organization of the ancestral insect mitochondrial genome (Cameron 2014). Phylogenetic analysis of protein-coding sequences yielded the same tree topology for the Syntermitinae subfamily as previously reported by Bourguignon et al. (2015) using partial mitochondrial genomes of *Labiotermes labralis* and *Embiratermes neotenicus* generated by PCR amplification and Illumina sequencing (Figure 1).

**Figure 1.**
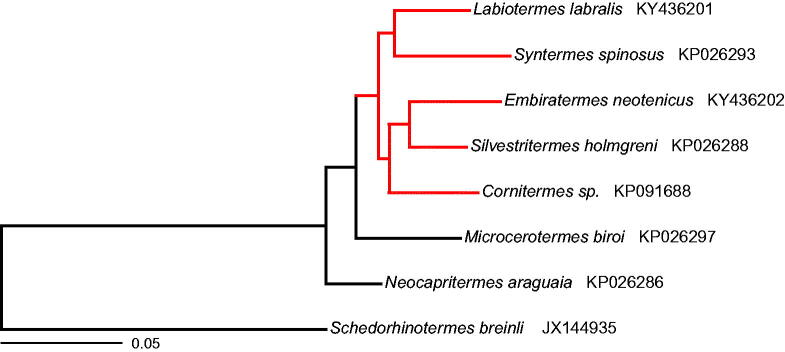
Maximum likelihood phylogenetic tree based on the amino acid sequences of all protein-coding genes of selected termite mitogenomes. The tree was built with RaxML version 8.2.9 (Stamatakis 2014). Members of the Syntermitinae subfamily are highlighted in red. GenBank accession numbers are given after the species name.

This study adds two complete mitochondrial genomes to the growing list of insect mitogenomes. Such extensions of mitogenome databases are important because they will increase the accuracy of future phylogenetic and evolutionary studies of arthropods, and improve the accuracy of high-throughput analyses in community ecology based on metagenomic datasets (Crampton-Platt et al. 2016).
